# The life-history trade-off between fertility and child survival

**DOI:** 10.1098/rspb.2012.1635

**Published:** 2012-10-03

**Authors:** David W. Lawson, Alexandra Alvergne, Mhairi A. Gibson

**Affiliations:** 1 Department of Anthropology, University College London, 14 Taviton Street, London WC1H 0BW, UK; 2 Department of Animal and Plant Sciences, University of Sheffield, Western Bank, Sheffield S10 2TN, UK; 3 Department of Archaeology and Anthropology, University of Bristol, 43 Woodland Road, Bristol BS8 1UU, UK

**Keywords:** parental investment, sibling competition, evolutionary demography, demographic transition, multi-level modelling

## Abstract

Evolutionary models of human reproduction argue that variation in fertility can be understood as the local optimization of a life-history trade-off between offspring quantity and ‘quality’. Child survival is a fundamental dimension of quality in these models as early-life mortality represents a crucial selective bottleneck in human evolution. This perspective is well-rehearsed, but current literature presents mixed evidence for a trade-off between fertility and child survival, and little empirical ground to evaluate how socioecological and individual characteristics influence the benefits of fertility limitation. By compiling demographic survey data, we demonstrate robust negative relationships between fertility and child survival across 27 sub-Saharan African countries. Our analyses suggest this relationship is primarily accounted for by offspring competition for parental investment, rather than by reverse causal mechanisms. We also find that the trade-off increases in relative magnitude as national mortality declines and maternal somatic (height) and extrasomatic (education) capital increase. This supports the idea that socioeconomic development, and associated reductions in extrinsic child mortality, favour reduced fertility by increasing the relative returns to parental investment. Observed fertility, however, falls considerably short of predicted optima for maximizing total offspring survivorship, strongly suggesting that additional unmeasured costs of reproduction ultimately constrain the evolution of human family size.

## 1. Introduction

Evolutionary anthropologists argue that human reproductive physiology and behaviour have evolved through a process of natural selection to maximize inclusive fitness, i.e. the production of long-term genetic descendants [1–3]. Maximizing inclusive fitness requires that somatic and extrasomatic capital be strategically allocated to competing dimensions of an organism's life history. Numerous life-history trade-offs have been described in the literature, including trade-offs between growth and reproduction [4,5], between reproduction and survival [6,7] and between quantity and quality of offspring [8,9]. Optimization of this latter quantity–quality trade-off is proposed to explain global diversity in human fertility (i.e. number of births), both within and between societies, including modern demographic shifts to below replacement fertility [2,10]. A key tenet of this perspective is that individuals adjust reproductive decisions depending on the trade-off between fertility and child survival, a crucial selective bottleneck in human evolution [9,11]. However, the current literature provides little empirical ground to evaluate the general importance of offspring quantity–quality trade-offs in human life history or to identify the role of socioecological and individual-level factors that influence the benefits of fertility limitation [10]. In this paper, we analyse comparative demographic survey data from sub-Saharan Africa to consider the evidence for a life-history trade-off between fertility and child survival, and quantify variation in this trade-off by both regional and maternal characteristics. Furthermore, we evaluate whether observed fertility rates in the developing world match with predicted optima for maximizing reproductive success, as measured by total number of surviving offspring.

### (a) Fertility, child survival and reproductive success in humans

Child survival is a critical component of reproductive success in traditional societies, with the large majority of deaths prior to adulthood occurring in the first 5 years of life [12]. In fact, owing to relatively low variance in female fertility, it has been suggested that differences in child survival, rather than in number of births, may have represented the primary determinant of reproductive success throughout human evolutionary history [9]. As such, we can expect the evolved physiological, psychological and cultural mechanisms that regulate fertility to be sensitive to the nature of child mortality risk. First, optimal fertility will depend on the extent to which energetic investments predict offspring survival. In traditional societies, child mortality risk is hypothesized to be relatively extrinsic, i.e. largely independent of variation in parental investment, ultimately favouring strategies of high fertility and relatively low parental care. This may be because of a range of socioecological factors that restrict parental ability to ensure offspring survival, such as unavoidably high pathogen loads, poor sanitation and healthcare access, and vulnerabilities to subsistence failure, natural disasters and violent conflict [13,14]. It has further been argued that the initial stages of demographic transition (i.e. the sequential decline in mortality and fertility commonly observed as populations undergo socioeconomic development) are characterized by a selective reduction in extrinsic child mortality, in turn triggering reductions in fertility by increasing the relative returns to parental investment [2,15,16]. Further reductions in fertility may then occur in response to emerging pay-offs to investments in education and heritable wealth as additional dimensions of offspring quality, which may or may not predict inclusive fitness in the long-term [2,10]. Second, optimal fertility will be modified by the level to which offspring are reliant on parents. For example, competition between siblings may be reduced when extended kin share the burden of childcare [17] or when modern welfare states safeguard base-level investments in child wellbeing [18]. In many societies, children themselves may also offset parental costs of child-rearing by directly contributing to subsistence activities or alloparental care [19]. However, the true extent to which variation in fertility in traditional populations, both generally and at the initial stages of the demographic transition, relates to changing costs of reproduction on offspring survival is poorly understood.

There is strong experimental evidence that increases in fertility have negative consequences for offspring survival in avian and mammalian species where parental investment is substantial [20]. Parallel research into human life history has produced mixed results. Some studies have found that higher fertility is associated with lower child survival [7,9,21,22], others have suggested that children in larger families are more likely to survive [23]; others report no relationship [13,24,25] and others still that relationships vary across population subgroups [26,27]. Only one study, to our knowledge, has concluded that observed fertility rates are optimal for maximizing reproductive success; with 83 per cent of women reported to maximize total number of offspring surviving to the age of 10 years [9].

Methodological limitations make it difficult to draw firm conclusions from this body of research [8,10,28]. On the one hand, non-experimental studies can underestimate trade-off functions because of phenotypic confounding; relatively wealthy or healthy mothers may be able to invest in both high reproductive rate and improved care of offspring, masking true costs of high fertility on offspring [29,30]. On the other hand, studies may overestimate the magnitude of trade-off effects by incorrectly attributing causality. A trade-off model implies that increased fertility causes reductions in child survival. This may occur when, for example, reproduction depletes somatic reserves required for healthy pregnancy or siblings compete for food, healthcare or direct supervision [31]. However, fertility–child survival relationships may also be established by ‘replacement’ or ‘insurance’ effects, whereby a mother has additional births to compensate for earlier infant death(s), or expected deaths in the face of predicted extrinsic mortality. Such reverse causal mechanisms are routinely considered by demographers [32,33], but have often been neglected in anthropological studies of human life history.

### (b) Aims of the current study

In this study, we examine the relationship between fertility and <5 years child mortality in data compiled from national demographic surveys for 27 sub-Saharan African countries. Sub-Saharan Africa currently not only has the highest child mortality and fertility rates in the world, but also encompasses considerable socioeconomic and demographic diversity, with most countries at early- to mid-stages of the demographic transition. By using large sample data, including comparative information on birth histories, household socioeconomics and maternal health, we aim to address several limitations of past research. First, we quantify fertility–child survival relationships in each country adjusting for differences in both maternal somatic and extrasomatic capital in an attempt to exclude potential phenotypic confounds. Second, we harness the unique comparative nature of our data to quantify both socioecological and individual-level variation in the benefits of fertility limitation. Specifically, we examine how the magnitude of the trade-off relates to variation in national-level mortality and to individual differences in age at reproduction and maternal somatic and extrasomatic capital. We then consider relationships between fertility and estimated reproductive success, comparing observed fertility rates with predicted optima for maximizing total offspring survivorship. Finally, we conduct supporting analyses to explore the extent to which relationships between fertility and child survival may be explained by reverse causal mechanisms whereby high child mortality causes high fertility.

## 2. Demographic data and methods

### (a) African Demographic and Health Surveys

The Demographic and Health Surveys (DHSs) are national demographic surveys, carried out throughout the developing world at regular intervals since the 1980s, and funded primarily by the United States Agency for International Development (www.measuredhs.com). Data collection is designed to facilitate cross-national comparison, with each survey conducted using a standardized questionnaire and protocol. Notably, however, sampling is focused on the household unit, a concept difficult to fully standardize in the face of considerable cultural variation in residence patterns [34]. For sampled households, all resident women aged between 15 and 49 years provide a complete birth history for all pregnancies resulting in a live birth, along with additional socioeconomic and health data. Our analyses use these data to model relationships between fertility and child survival, taking into account its nested three-level structure; births (level 1) clustered within mothers (level 2), which are in turn clustered within countries (level 3). We include the most recently available sub-Saharan African DHSs with appropriate data. In total, this amounts to 27 countries, surveyed between 2003 and 2008 (see the electronic supplementary material, table S1). Contraceptive use is low in sub-Saharan Africa during this period, with only 18 per cent of married women using modern contraceptives, compared with 63 per cent in Latin America and 61 per cent in Asia [35]. In 2006, total fertility rates ranged from a high of 7.3 (Niger) to a low of 3.3 births per woman (Namibia and Zimbabwe).

### (b) Statistical analysis

Past studies of the quantity–quality trade-off have used standard regression techniques and focused analysis at the mother level, estimating the relationship between the completed fertility of post-menopausal women and total offspring survivorship. This method is sensitive to recall bias when based on retrospective birth histories, ultimately underestimating the number of child deaths. Focusing on post-menopausal women also biases the sample towards healthier mothers in high-mortality populations. Our analysis uses multilevel logistic regression (binomial error structure) and is focused at the child level; estimating the relationship between fertility *at the time of survey* for women across the full reproductive age range (i.e. 15–49 years) and the odds of survival for *selected past births* (see below). A multi-level approach corrects for statistical biases inherent in hierarchical data and enables direct estimates of residual variation in child survival associated with each level [36]. This makes it possible not only to reliably estimate the overall relationships between fertility and child survival, but also to assess the moderating influence of contextual effects (i.e. mother and country) on this relationship. Our approach is equivalent to past studies using completed fertility in that child survival may be influenced by both older and younger siblings and those that are living, deceased or not yet born at the time of the child's death. Even in the latter case, resource competition may contribute to child deaths as the impending birth of younger siblings may be associated with a range of adverse maternal behaviours required to achieve a high fertility rate (e.g. early termination of breastfeeding, simultaneous pregnancy and care of young children).

To ensure that maternal and household characteristics correspond as closely as possible to the timing of child deaths, and to limit recall bias of less recent births and deaths, we restrict our analysis to predicting the survival of children born within 5–10 years prior to the survey date (children must be born 5+ years ago to estimate survival to this age with logistic regression). Limiting analysis to recent births also ensures that children of older women relative to those of younger women are not disproportionality represented in the sample, which would bias estimates against the latter [37]. To account for phenotypic correlations, we include two measures of extrasomatic capital: a household wealth index and maternal education, and one measure of somatic capital: maternal height (see the electronic supplementary material, table S2 for descriptives on all covariates). The wealth index is based on ownership of items such as a radio, television, refrigerator; and multiple indicators of housing quality and available facilities. It is standardized for each country to a mean of 0 and a s.d. of 1, then banded into quintiles. Maternal height is selected as a measure of somatic capital. Unlike alternative measures, such as body mass index, height remains fairly fixed over the reproductive lifespan with limited potential to be influenced by prior childbearing or current pregnancy.

Our approach enables a robust comparative analysis of individual and socioecological variation in the life-history trade-off between fertility and child survival. In particular, multi-level modelling enables the estimation of individual-level coefficients across groups, accounting for group-level variation in the uncertainty of individual-level coefficients, and estimation of regression coefficients for particular groups [38]. Specifically, in our analysis, we first consider that the odds of child survival can vary by country by including a random intercept for the identity of the countries in our sample. Second, we consider that the relationship between fertility and child survival might vary across countries by including a random slope for the effect of fertility on child survival. Varying slopes can be interpreted as interactions between an individual predictor (i.e. women's fertility) and group indicators (i.e. countries) [38]. We then investigate whether there is a correlation between the random intercepts and the random slopes, i.e. whether the effect of fertility on child survival depends on the level of child survival estimated for each country. To do so, we perform a likelihood ratio test comparing two models differing only by the inclusion of a correlation coefficient between the random intercepts and slopes. We also test for interaction effects between individual-level coefficients for fertility and specific maternal characteristics acting as proxies for maternal somatic and extrasomatic capital (as described earlier). All analyses were carried out in R v. 2.11.1, using the package ‘lme4’ [39] and ‘arm’ [40], with parameters estimated using the restricted maximum-likelihood method.

## 3. Results

In total, our sample includes 163 827 births and 101 195 mothers. Sampled births are evenly split by gender, 22 per cent were firstborns, 34 per cent second or third born, 44 per cent fourth born or later. Of those children second or later born, 14 per cent had a preceding birth interval of 18 months or less, 51 per cent between 18 months and 3 years, and 35 per cent 3 years or more. Ninety-six per cent of births were singletons, with the remaining 4 per cent being multiple births. The overall odds of child survival for sampled births is 5.9 (i.e. one death for every 5.9 children surviving to their fifth birthday), ranging at the country level between 3.6 (Mali) and 15.9 (Zimbabwe).

### (a) The trade-off between fertility and child survival

**Figure 1.**
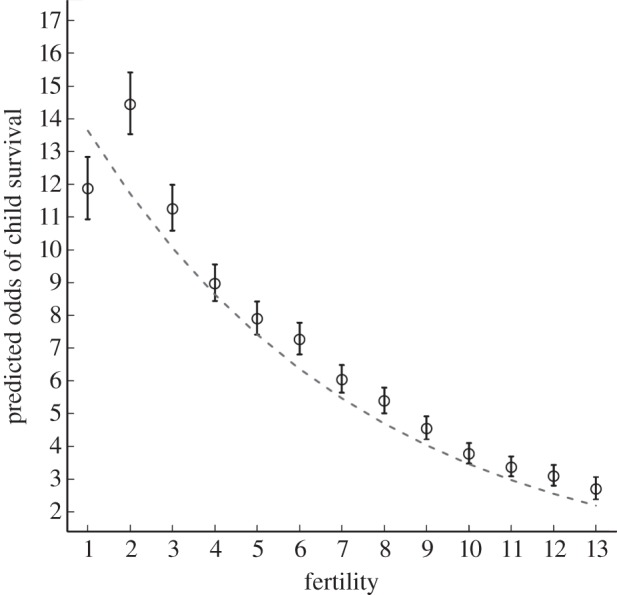
Fertility and predicted odds of child survival. As fertility increases, the odds of child survival to age 5 years decreases. Predicted values are adjusted for maternal age at birth of child, height, educational level, household wealth, marital status and urban versus rural residence (see the electronic supplementary material, table S3). The dashed line and points plot the effect of fertility entered as a linear and categorical term respectively.

Adjusting for maternal characteristics (i.e. age at birth of child, height, education, wealth and marital status) and rural versus urban residence, the effect of fertility is substantial and significant; for every additional maternal birth, the odds of survival for each child decrease by 14 per cent (ExpB = 0.86, 95% confidence interval (CI): 0.85–0.87, *p <* 0.001; electronic supplementary material, table S3). Figure 1 displays the fertility–child survival relationship from this fully adjusted model with fertility entered as a linear vs. categorical term. Comparing these functions confirms that the relationship approximates a linear function, with odds of survival to age 5 years declining with each additional maternal birth. The only notable deviation from linearity is that children with no siblings are less likely to survive than children with one sibling, reflecting the well-known higher mortality risks of first-born offspring [41].

In addition to fertility, child survival is influenced by maternal characteristics. Children born to younger mothers were less likely to survive. For example, children born to mothers of under 16 years experience 67 per cent lower odds of survival (ExpB = 0.33, 95% CI = 0.31–0.36, *p* < 0.001) compared with children born to mothers between the ages of 24–31 years. Child survival also increased with both somatic and extrasomatic capital. Children of mothers in the tallest quartile have 26 per cent (ExpB = 1.26, 95% CI = 1.21–1.32, *p* < 0.001) higher odds of survival than children of mothers in the shortest quartile. Secondary relative to no education increases odds of child survival by 39 per cent (ExpB = 1.39, 95% CI = 1.31–1.48, *p* < 0.001), and being from the wealthiest rather than poorest quintile of the population increases odds of child survival by 38 per cent (ExpB = 1.38, 95% CI = 1.29–1.47, *p* < 0.001). Additionally, children of formerly or never married mothers have 25 per cent (ExpB = 0.75, 95% CI = 0.71–0.79, *p* < 0.001) and 26 per cent (ExpB = 0.74, 95% CI = 0.65–0.83, *p* < 0.001) lower odds of survival, respectively, compared with children of married mothers. Finally, children born in rural areas have 7 per cent (ExpB = 0.93, 95% CI = 0.89–0.97, *p* < 0.01) lower odds of survival compared with children born in urban areas. The fully adjusted effect of fertility is actually slightly smaller than the effect of fertility unadjusted for differences in maternal capital, i.e. 14 per cent versus 16 per cent reduction in odds of child survival per additional birth respectively (see the electronic supplementary material, table S3). Thus, there is little indication that phenotypic correlations mask the costs of reproduction in these data.

### (b) Socioecological and individual-level variation in the trade-off

**Figure 2.**
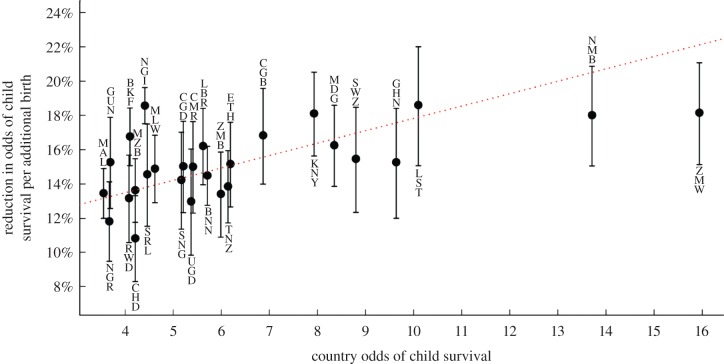
Socioecological variation in the trade-off between fertility and child survival. Increasing fertility leads to larger reductions in the odds of survival to age 5 years in countries where child survival is relatively more common overall (*r* = −0.74, *p* < 0.001, electronic supplementary material, table S3). Displayed confidence intervals are set at 95%. BNN, Benin; BKF, Burkina Faso; CMR, Cameroon; CHD, Chad; CGB, Congo–Brazzaville; CGD, Congo Democratic Republic; ETH, Ethiopia; GHN, Ghana; GUN, Guinea; KNY, Kenya; LST, Lesotho; LBR, Liberia; MDG, Madagascar; MLW, Malawi; MAL, Mali; MZB, Mozambique; NMB, Namibia; NGR, Niger; NGI, Nigeria; RWD, Rwanda; SNG, Senegal; SRL, Sierra Leone; SWZ, Swaziland; TNZ, Tanzania; UGD, Uganda; ZMB, Zambia; ZMW, Zimbabwe.

We find a strong signal of socioecological variation in the effect of fertility on child survival. Including a country-level random effect for fertility substantially improves model fit (*χ*^2^_1_ = 52.32, *p* < 0.001; electronic supplementary material, table S3). Each additional maternal birth reduces the odds of survival to age 5 years by 11 per cent in Chad (ExpB = 0.89, 95% CI = 0.87–0.92, *p* < 0.001) rising to 19 per cent in Lesotho (ExpB = 0.81, 95% CI = 0.78–0.85, *p* < 0.001). Model fit is also further improved by including a correlation between the country odds of survival (i.e. the random intercept) and the effect of fertility (i.e. the random slope; *χ*^2^_1_ = 11.93, *p* < 0.001; electronic supplementary material, table S3). This correlation is strongly negative (*r* = −0.739), implying that as the odds of survival increase, the effect of fertility on survival becomes more negative. Thus, the trade-off between fertility and child survival is of larger relative magnitude in countries where child survival is more likely overall. Figure 2 illustrates this trend, plotting, for each country, the mean odds of survival for sampled births against the estimated reduction in the odds of survival per additional maternal birth.

**Figure 3.**
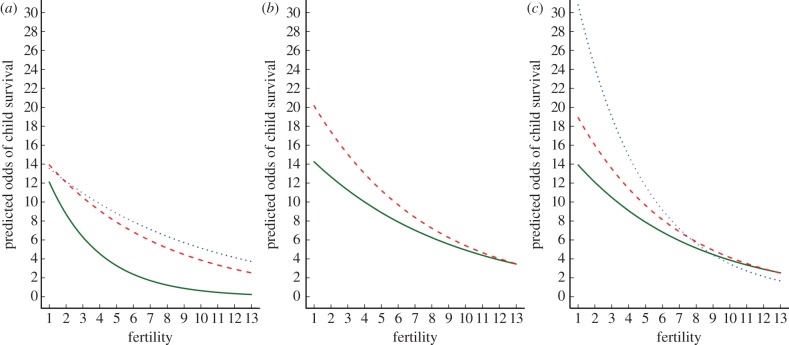
Individual variation in the trade-off between fertility and child survival by maternal (*a*) age at survey (dotted line, 40+ years; dashed line, 30–34 years; solid line, <25 years), (*b*) height (dashed line, tallest; solid line, shortest), and (*c*) educational attainment (dotted line, secondary education; dashed line, primary education; solid line, no education). Mothers with high fertility at a young age, tall mothers and well-educated mothers all face stronger trade-offs between fertility and child survival to age 5 years. Predicted values are based on fully adjusted models for mothers with mean values on all other characteristics (i.e. aged 30–34 years at survey, between 24 and 31 years at the birth of the child, in the second height quartile, middle wealth quintile, no education, married and a rural resident) (see the electronic supplementary material, table S4).

Further analysis also reveals that the strength of the relationship between fertility and the odds of child survival is predicted by maternal characteristics, independently of overall mortality rate (figure 3). First, fertility has a larger impact on child survival for mothers surveyed at relatively younger ages, most likely owing to short birth spacing. For example, assuming mean values for all other maternal characteristics, each additional birth reduces the odds of child survival to age 5 years by 28 per cent for mothers under 25 years old (ExpB = 0.72, 95% CI = 0.69–0.76, *p* < 0.001), but only 10 per cent for mothers of 40 years or older (ExpB = 0.90, 95% CI = 0.85–0.94, *p* < 0.001). Second, despite higher odds of child survival overall, relatively tall and well-educated mothers experience larger relative costs of increasing fertility. For example, assuming mean characteristics on all other traits, each additional birth reduces the odds of child survival by 9 per cent for mothers in the shortest quartile (ExpB = 0.91, 95% CI = 0.87–0.96, *p* < 0.001), but by 12 per cent for mothers in the tallest quartile (ExpB = 0.88, 95% CI = 0.84–0.93, *p* < 0.001); while each additional birth reduces the odds of child survival by 11 per cent for mothers with no education (ExpB = 0.89, 95% CI = 0.85–0.93, *p* < 0.001), compared with 20 per cent for mothers with a secondary education (ExpB = 0.80, 95% CI = 0.77–0.84, *p* < 0.001). As such, the survival advantage of children of tall and well-educated mothers appears conditional on relatively low fertility (figure 3). An additional interaction term also indicates that the costs of high fertility are weaker in the richest families. However, this interaction is small in magnitude and significant only in the presence of the larger interaction between fertility and education, confirming that, in general, improved extrasomatic capital increases the relative costs of reproduction (see the electronic supplementary material, table S4).

### (c) Fertility and predicted reproductive success

**Figure 4.**
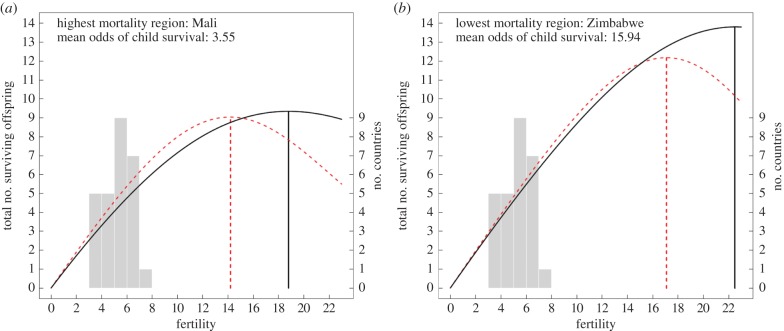
(*a*,*b*) Fertility and predicted reproductive success by mortality rate and maternal capital (solid line, low maternal capital; dashed line, high maternal capital). Optimal fertility for maximizing predicted reproductive success (total number of children surviving to age 5 years) varies between and within countries. Observed fertility rates fall considerably short of calculated optima everywhere. Predicted values are based on fully adjusted models and assumed women are married and at the mean values for age at survey and birth of child (see the electronic supplementary material, table S4). Low capital mothers: women in the shortest height quartile, with no education, in the poorest wealth quintile and rural resident. High capital mothers: women in the tallest height quartile, secondary education, in the richest wealth quintile and urban resident. Overlaid histograms represent the total fertility rates for all 27 countries included in the analysis (see the electronic supplementary material, table S1).

Figure 4 shows the relationship between fertility and predicted reproductive success (total number of children surviving to the age of 5 years) for mothers in countries with the highest and lowest child mortality risk (i.e. Mali and Zimbabwe, respectively), and at the estimated extremes of maternal capital within those countries. Predicted reproductive success is based on fully adjusted models (see the electronic supplementary material, table S4) and calculated as fertility multiplied by the derived probability of survival to age five years for children of mothers at fertility level, assuming that the mother is married and at the mean age at survey (i.e. 30–34 years). For illustrative purposes, we calculate predicted reproductive success for fertility to over 20 births, assuming that the effect of fertility on child survival retains a linear relationship beyond observed data (<5% of sampled mothers had over 10 births). For ‘low capital mothers’, we estimate relationships assuming women are in the shortest height quartile, with no education, in the poorest wealth quintile and resident in rural areas. For ‘high capital mothers’, we estimate relationships assuming women are in the tallest height quartile, have secondary education or above, are in the richest wealth quintile and are resident in urban areas. This model should be considered as narrowly parametrized in the sense that the estimated extremes of local mortality are based only on national values, and the extremes of maternal capital are based only on the covariates included in our model.

Our results illustrate that reproductive success depends not only on the effect of fertility on child survival (i.e. the trade-off function), but also on the child mortality risk for a given woman and local socioecology. For example, the odds of child survival are over four times greater in Zimbabwe than in Mali. This difference is so large that, despite a stronger trade-off function for Zimbabwean mothers (figure 2), they are always predicted to achieve a higher reproductive success than Malian women at any given number of births. Within both Mali and Zimbabwe, mothers of high capital also achieve a higher reproductive success at most fertility levels. However, at this level, the stronger trade-off function for high capital mothers (figure 3) means that their advantage eventually wanes, leading to a lower predicted optimum fertility. Thus, in Mali, high capital mothers are predicted to maximize reproductive success at around 14 births, whereas low capital mothers continue to increase reproductive success with increasing fertility up until around 19 births. The steeper trade-off function for high capital mothers also implies that at the extremes of high fertility they would achieve lower reproductive success than low capital mothers. However, at this point, predicted relationships become extrapolated beyond observed data. Given the sequential nature of human births and that most child deaths occur in the first few years of life, we might anticipate limited scope for the survival of earliest births to be influenced by investment competition with later born children. Thus, it is more likely that reproductive success will plateau rather than decline at very high fertility (see also Meij *et al*. [22]).

Figure 4 demonstrates that predicted fertility optima for maximizing reproductive success fall substantially higher than observed fertility for all countries in our sample (overlaid histogram on figure 4), and across traditional populations more generally; where total fertility rates are rarely recorded beyond eight births per woman [42]. This suggests that sibling competition for early survival, at least in isolation, is insufficiently costly to account for why women limit the pace of reproduction even in the highest fertility populations.

### (d) Does high fertility cause high child mortality?

**Figure 5.**
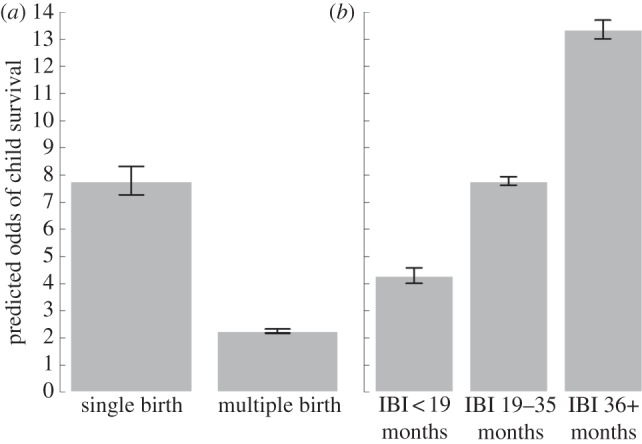
(*a*,*b*) Twinning, inter-birth intervals (IBI) and child survival. Short birth intervals and twinning are associated with reduced odds of survival to age 5 years. Predicted values are based on fully adjusted models and for mothers with mean values on all other characteristics (i.e. aged 30–34 years at survey, between 24 and 31 years at the birth of the child, in the second height quartile, middle wealth quintile, no education, married and rural resident; electronic supplementary material, table S4).

The analyses above assume that observed relationships are driven by high fertility causing high child mortality. But could the reverse also be true? We undertook supporting analyses to address this question. The odds of survival are substantially reduced even when we exclude the effect of subsequent births on child survival (i.e. excluding the possibility that births are caused by previous deaths), which strongly suggests that replacement births cannot be the primary driving force underlying observed relationships. Adjusting for number of younger siblings, fertility reduces odds of child survival by 10 per cent for each additional birth (ExpB = 0.90, *p* < 0.001; electronic supplementary material, table S4). Hypothetical insurance strategies, whereby fertility is increased to compensate for predicted extrinsic mortality (i.e. largely unavoidable or care-independent deaths), also provide poor fit to the data. The costs of high fertility are not weakened by the exclusion of vulnerable neonatal deaths (ExpB = 0.86, 95% CI = 0.85–0.87, *p* < 0.001; 30% of deaths occurred within one month of birth), which are arguably most likely to be caused by extrinsic factors in environments where mothers have restricted ability to seek treatment for birth complications or infant infections. Furthermore, as we demonstrate, fertility–child survival relationships actually increase in magnitude for healthy and well-educated mothers who are less likely to be exposed to extrinsic sources of child mortality. Finally, we show that, independent of fertility, tight birth intervals and twinning hold strong negative associations with child survival (figure 5), most parsimoniously explained by competition between offspring for parental investment.

## 4. Discussion

### (a) The life-history trade-off between fertility and child survival

The idea that variation in fertility can be understood as the optimization of life history is central to evolutionary accounts of human behaviour [1–3]. The analyses presented here provide empirical support for the predicted life-history trade-off between quantity and quality of offspring; with each additional maternal birth decreasing the odds of child survival in all countries. We also provide, to our knowledge, the first demonstration that the magnitude of this trade-off varies both between and within socioecological contexts, ultimately contributing to distinct fertility optima. Our results illustrate that optimal fertility for maximizing reproductive success is contingent on both local risk of child death and the effect of fertility on that risk, i.e. the trade-off function. The relative costs of high fertility on child survival, as estimated by the percentage reduction in odds of child survival per additional birth, are largest in low-mortality countries and where maternal somatic (height) and extrasomatic (education) capital is high. Trade-offs are also stronger for mothers who attain high fertility at a relatively young age. As such, increasing costs of high fertility on offspring survival and related later outcomes may contribute to perceived or real benefits of reduced and delayed fertility observed as populations undergo socioeconomic development. In addition, our findings help us to explain failure of many past studies to detect costs of high fertility on child survival [13,23–25]; as weak trade-off effects may be difficult to detect in populations where maternal condition is poor or births widely spaced. However, while negative correlations between fertility and child survival are strong at the national level (see the electronic supplementary material, table S1), relatively few child deaths appear to be caused by high fertility. Thus, we conclude that fertility limitation does not maximize reproductive success across the extensive socioecological range of our sample. This suggests that additional fitness costs of reproduction, such as later competition between surviving offspring or masked physiological costs of offspring production, ultimately constrain the evolution of human family size.

It is perhaps unsurprising that high fertility accounts for only a small proportion of child deaths. Given high energetic costs of parental investment, natural selection favours well-established physiological mechanisms that prevent conception and birth when maternal condition dictates a low chance of offspring survival [43]. Consequently, we can expect births to primarily occur when child survival either is highly likely or unavoidably stochastic. Our results imply the latter situation characterizes fertility patterns in much of the developing world in the face of high extrinsic mortality risks. For example, two-thirds of child deaths in the developing world are caused by infectious diseases such as malaria, pneumonia and diarrhoea, difficult to prevent in the absence of modern sanitation and medical care [44]. Under such conditions, limiting fertility as a strategy to increase levels of parental investment is more likely to be reduce reproductive success. This does not contradict the observation that human offspring are highly dependent on parental investment, but rather implies that above a readily obtainable threshold, variance in levels of parental care is poorly predictive of mortality [13,14]. Our results also support the hypothesis that socioeconomic development leads to a selective reduction in such extrinsic sources of mortality, ultimately magnifying the relative pay-offs to fertility limitation [2,15,16]. This helps us to explain the close sequential association between mortality and fertility decline that characterizes the initial stages of demographic transition. Mothers of high extrasomatic capital benefit most from fertility limitation, perhaps because they achieve greater access to the institutional and technological advancements that reduce extrinsic sources of child mortality. Maternal education, for example, is associated with improvements in effective sanitation and hygiene practice [45]. A number of recent studies have also presented evidence that socioeconomic development leads to emerging pay-offs to fertility limitation on offspring health [46], education [16,47,48] and socioeconomic mobility [49,50]. These additional benefits may ultimately motivate further reductions in fertility in later stages of the demographic transition [2,10].

Even in the highest mortality country in our sample, where almost one in four children die by age 5 years, observed fertility rates fall dramatically short of estimated optima for maximizing reproductive success. This conclusion is shared by studies of both hunter–gatherer and traditional agriculturalist populations, where estimated trade-offs relationships are either weak [21,22], or child survival unrelated to fertility or number of living siblings [13,24,25]. Only in the Dogon of Mali has evidence been presented that observed fertility maximizes total number of surviving offspring [9]. Our analyses suggest the Dogon are unusual in combining an overall high child mortality rate with a strong trade-off function (each additional child in the family increases the odds of child death by 26% [51]). However, the Dogon study, while reliant on a relatively small sample size, is also unusual in its inclusion of a range of covariates and prospective design [9,28]. Future studies need to match such methodological sophistication to determine the extent to which the Dogon may be unusual, particularly in the context of high-mortality environments. A limitation of our study is its reliance on retrospective birth histories. It is also true that mortality patterns in contemporary sub-Saharan Africa may not correspond well to those experienced in ancestral environments. Nevertheless, current literature and results of the present analysis imply that sibling competition for child survival generally presents a modest selective constraint on human fertility.

### (b) Have we underestimated the trade-off?

It is possible that the discrepancy between observed and predicted optimal fertility can be accounted for by an underestimation of the trade-off. Unlike many previous studies, we controlled for differences in maternal extrasomatic and somatic capital in estimating the effect of fertility on child survival. Further unmeasured factors, such as levels of allomaternal support [52] or genetic advantages [22], which simultaneously favour increased investment in offspring survival and reproductive rate may partially mask the costs of high fertility. However, we found that improved maternal capital increased child survival, but was not associated with higher fertility (see the electronic supplementary material, table S2), and that adjustment for differences in maternal capital did not increase the estimated trade-off. This suggests that confounding relationships are of relatively minor importance in our sample.

Considering only a single generation may also underestimate the full cost of high fertility. For example, high fertility may reduce investment in grandchildren, either because it is traded-off against post-reproductive survival [7], or because available grandparental investment is diluted across additional grandchildren [53]. By considering only living mothers, we also exclude child survival costs stemming from maternal mortality at childbirth. However, such additional costs of high fertility would need to be considerable in order to account for observed fertility rates. Indeed, our study is perhaps more likely to have overestimated the strength of the trade-off because we estimate relationships assuming causality runs only from fertility to child survival, rather than the reverse direction. Supporting analyses indicate that the negative association between fertility on child survival is unlikely to be accounted for by hypothesized insurance or replacement births. However, excluding such mechanisms is difficult without experimental data. Future studies need to explicitly demonstrate that a dilution of parental investment drives estimated costs of high fertility on child survival. Such studies are also rare with regard to alternative dimensions of offspring quality, including negative effects of large sibship size on educational attainment in Western populations [31,54].

### (c) Why is high fertility so low?

If not competition for survival, what additional costs of reproduction set the upper limits of high fertility? One possibility is competition between surviving offspring for later reproductive opportunities. For example, several studies have shown that fertility is negatively associated with offspring height [46,55], which may ultimately reduce adult reproductive success in both sexes [56]. Yet, current literature suggests that siblings only depress marital and reproductive success in situations where material wealth is transferred across generations, i.e. stemming from the development of agriculture or wage–labour [10,57]. For daughters (the non-inheriting sex) and for families who do not transfer wealth, the presence of siblings promotes higher reproductive success, a likely consequence of beneficial cooperative coalitions [24,57]. In fact, hunter–gatherers, who lack material wealth transfers, typically have lower fertility than agriculturalists [58], suggesting that adult sibling competition does not necessarily diminish fertility. A second possibility is that inter-generational competition over shared resources available for reproduction may establish additional selective pressure on fertility limitation not accounted for by optimization models conceptualized at the individual-level. This form of reproductive conflict has recently been proposed to account for the evolution of menopause [59], but could also be applied to the evolution of family size.

Finally, in the absence of intense competition between offspring for post-natal investment, fertility in pre-demographic transition societies may remain primarily determined by availability of somatic reserves required for offspring production in pregnancy. Such costs may be largely masked when studying life-history trade-offs with observational rather than experimental data. As such, ‘natural fertility’ may be better studied in terms of energetic trade-offs between fertility and somatic maintenance and the related physiological constraints on reproduction [43]. Ultimately, this can also be conceptualized as a quantity–quality trade-off, but with the relevant competition being between (hypothetical) offspring for pre-natal investment, rather than for post-natal investment among those offspring born. This conclusion has some consistency with classic theoretical models of the demographic transition, which assume psychological and cultural mechanisms governing the conscious regulation of fertility emerged relatively recently in human history [60–62]. Lloyd & Ivancov [62], for example, describe the demographic transition as a shift from ‘family building by fate’ to ‘family building by design’. These considerations suggest that future research will need to integrate optimality modelling with an improved understanding of the mechanistic constraints that regulate reproduction if we are to achieve a satisfying evolutionary account of human fertility.
